# Implications of ACMG guidelines to identify high-risk acute lymphoblastic leukemia patients with hereditary cancer susceptibility syndromes (HCSS) in a highly consanguineous population

**DOI:** 10.1186/s12887-021-02749-2

**Published:** 2021-06-16

**Authors:** Sara Aslam, Shabana, Mehboob Ahmed

**Affiliations:** Institute of Microbiology and Molecular Genetics, University of the Punjab, Lahore, 54590 Pakistan

**Keywords:** Consanguinity, Hereditary cancer susceptibility syndrome (HCSS), Risk assessment, ACMG guidelines, Acute lymphoblastic leukemia (ALL)

## Abstract

**Background:**

Hereditary cancer susceptibility syndrome (HCSS) contributes to the cancer predisposition at an early age, therefore, identification of HCSS has found to be crucial for surveillance, managing therapeutic interventions and refer the patients and their families for genetic counselling. The study aimed to identify ALL patients who meet the American College of Medical Genetics (ACMG) criteria and refer them for the genetic testing for HCSS as hereditary leukemia and hematologic malignancy syndrome, and to elucidate the significance of high consanguinity with the prevalence of inherited leukemia in Pakistani population.

**Methods:**

A total of 300 acute lymphoblastic leukemia patients were recruited from the Children’s Hospital, Lahore, Pakistan from December 2018 to September 2019. A structured self-reporting questionnaire based on family and medical history of the disease was utilized for the data collection.

**Results:**

In our cohort, 60.40% of ALL patients were identified to meet ACMG criteria. Among them, a large number of patients (40.65%) solely fulfil the criteria due to the presence of parental consanguinity. However, parental consanguinity showed protective impact on the onset at early age of disease [OD = 0.44 (0.25–0.77), *p*-value = 0.00] while, a family history of cancer increased the risk of cardiotoxicity [OD = 2.46 (1.15–5.24), *p*-value = 0.02]. Parental consanguinity shows no significant impact on the family history of cancer and the number of relatives with cancer.

**Conclusions:**

More than 50% of the ALL patients were considered the strong candidates’ for genetic testing of HCSS in the Pakistani population, and parental consanguinity was the leading criteria fulfilled by the individuals when assessed through ACMG guidelines. Our study suggests revisiting ACMG guidelines, especially for the criterion of parental consanguinity, and formulating the score based criteria based on; genetic research, the toxicology profile, physical features, personal and family history of cancer for the identification of patients for the genetic testing.

## Background

According to the global cancer observatory 2018, Asia is the most leukemia burdened region in the world with an incidence of 48.7% and a mortality rate of 53.7% of overall cases reported worldwide [1]. In Pakistan, acute lymphoblastic leukeima (ALL) is a predominant childhood cancer with an incidence of 20.8% as reported by the Punjab cancer registry 2017 [2]. Remarkable progress has been observed in improving the survival rate of acute lymphoblastic leukemia due to the implementation of the risk-adapted therapy and a greater understanding of the biological heterogeneity of the disease [3]. However, the etiology of leukemia is still unknown, and the major cause reported for this cancer is the same as other cancers involving the interaction of environmental factors and genetic susceptibility [4–7]. Hereditary cancer susceptibility syndromes include the predisposition to leukemia, often at an early age, caused by inherited mutations or polymorphisms. The identification of the HCSS aids in cancer surveillance and screening, optimizing the therapeutic response and advising the patient for genetic counselling to reduce the risk of cancer. The outcome of these practices helps to decline the incidence and morbidity rate of inherited leukemia among children [8, 9]. It is estimated that 5–10% of leukemia cases are attributed to genetic susceptibility [10]. However, Knapke et al., reported 29% of the survivors of childhood cancer to have HCSS and due to the presence of the familial history of cancer [11]. The assessment of hereditary cancer susceptibility syndromes (HCSS) as hereditary leukemia and hematologic malignancy syndromes was found crucial for patients with familial history of cancer in first and second-degree relatives [12].

Based on the evidence, early onset of cancer [13, 14], family history of cancer especially in a first and second degree of relatives [15], racial or ethnic differences [16] and consanguinity [17] are considered to be the major risk factors of hereditary cancer, and are included in the various criteria established for the identification of HCSS, formulated by different consensus groups to identify the patients and families at risk of developing cancer and refer them for genetic counselling [18]. The guidelines provided by the American College of Medical Genetics (ACMG) utilize consanguinity along with the familial history of cancer [19]. Apart from one study conducted in the highly consanguineous population of Saudi Arabia, data on the contribution of consanguinity to hereditary leukemia and hematologic malignancies syndromes are lacking. Consanguineous marriages are practised around the world and reported to have severe implications if practised in consecutive generations [20]. In Europe and America, the rate of consanguinity is reported to be less than 1%, and in Arab countries the rate is 20–50% [21] whereas, in Pakistan, it is reported to be 60% [22]. Among these populations, the most predominant degree of relatedness is the first cousins [20]. In adults, constitutional MMR deficiency (CMMRD) syndrome has also been identified in the high consanguinity population, characterised by haematological malignancies and brain tumour, however; its prevalence in paediatric patients is underreported [23, 24].

The present study aimed to estimate the ALL patients to refer for genetic testing of HCSS and to assess the role of consanguinity in the identification of HCSS in the highly consanguineous population of Pakistan.

## Materials and methods

### Study cohort and research ethics

The cross-sectional study was conducted at the University of the Punjab, and the Research Ethics and Biosafety Committee of University of the Punjab approved the study protocol related to the data collection from the human patients. The research was conducted in accordance with the Declaration of Helsinki. The study population comprised of 300 acute lymphoblastic leukemia patients (BCP ALL and T-ALL) aged 15 years or younger diagnosed from December 2018 to September 2019. Informed consent was obtained from the parents or legal guardians of all patients as; the age of participants was under 16 years. Data related to the personal information of patients and the medical history of the disease was obtained. The exclusion criterion included any other type of leukaemia and patients seropositive for infectious diseases. The first-degree relatives refer to the parents and siblings of the patients, second-degree relative refer to the half-sibling, grandparents, uncle, aunt, niece and nephew and third-degree relatives refer to other cousins and great grandparents of the affected child.

### Data collection

The data was collected from the Children’s Hospital, Lahore, Pakistan by using a subjective self-reporting questionnaire. The standardized face to face interviews was conducted to collect data regarding the family history of the disease. Patient charts were consulted to obtain data related to the age of the patient, gender, number of primary cancer and associated disorders. The complete blood cell count, echocardiogram and ultrasound reports were consulted for the WBC count, hepatosplenomegaly and cardiotoxicity. The treatment regimens of the ALL patients differ for standard-risk group (Age < 10 and WBC < 50,000 count) and high-risk group (Age > 10 and WBC ≥ 50,000 count). In the induction phase of the UKALL 2003 protocol, the standard-risk group were administered three drugs (vincristine, L-asparaginase and dexamethasone) and additional anthracyclines were administered to the high-risk group. The echocardiogram reports of the patients were considered that were conducted 15 days after the exposure of the patients with anthracycline chemotherapy and ultrasound reports were considered that were conducted 15 days after the completion of induction therapy. The flow cytometry reports (FCM) were assessed for the presence of Pre T-cell ALL (T-ALL) and B-cell precursor ALL (BCP ALL) markers and genetic screening reports for Ph-positive ALL (BCR/ABL1) of the patients were recorded. The patients who had a positive family history were interviewed further regarding the degree of family history with cancer and the number of relatives with cancer.

### Assessment of HCSS and statistical analysis

The assessment of patients for genetic testing of HCSS among the target population was done according to the guidelines provided (Table 1) by American College of Medical Genetics (ACMG) and Genomics and the National Society of Genetic Counselors (NSGC) [19]. It includes comprehensive personal and family history criteria that help in identification of high-risk individuals and referral for genetic counselling. A subgroup of family history and parental-consanguinity was considered to assess their impact on risk group parameters (age, WBC count), toxicity profile (hepatosplenomegaly, cardiotoxicity) and subtypes of ALL. The data were presented in the form of percentages, and chi-square test was employed to assess the categorical variables. The logistic regression model was used to assess the association of family history and familial consanguinity. All the models for the statistical analysis were expressed in the form of odds ratios (OR) and 95% confidence interval (CI). The level of significance was kept < 0.05 and IBMSPSS software was utilized for statistical analysis.

**Table 1:** ACMG/NSGC criteria for the assessment of hereditary cancer predisposition in leukemia

Diagnosis of leukemia < 18	Consanguineous parents
	Family history of LS-associated cancers
	Second primary cancer
	Sibling with a childhood cancer
Leukemia one additional Li-Fraumeni syndrome (LFS) tumor	the same patient or in 2 close relatives, one dx at age ≤ 45

## Results

### Patient characteristics and identification of HCSS

**Fig. 1:**
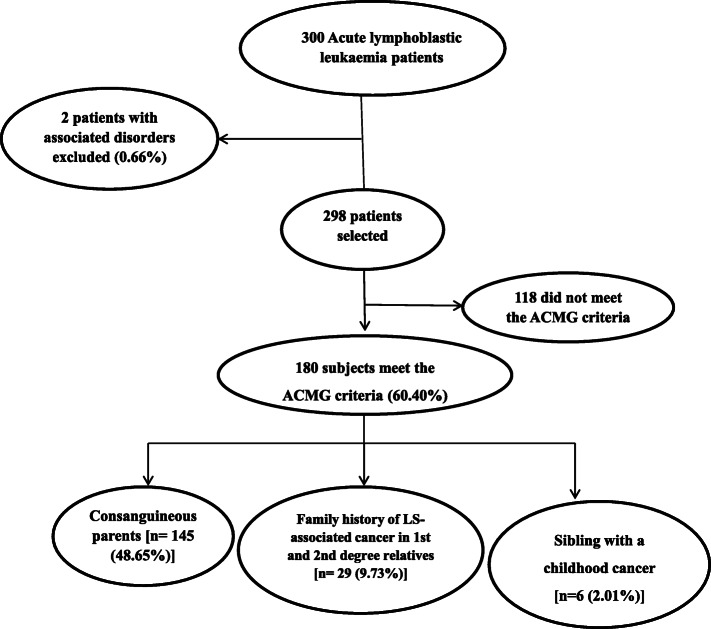
Consort diagram showing the selection of ALL patients with HCSS by using ACMG guidelines

In the present study, three hundred patients of acute lymphoblastic leukemia (ALL) were recruited for the study and among them, 2 (1.66%) were excluded due to the presence of underlying disorders/syndromes i.e. Down syndrome (Fig. 1). The mean age at the time of diagnosis was 6.62 ± 3.5 and the male to female ratio was 2.59:1. Parental consanguinity was 151 (50.67%) and familial history was 66 (22.14%) present in patients. In our cohort, 257 (86.24%) patients had BCP ALL and 41 (13.75%) patients had T-ALL. Only one patient had more than one primary cancers and it includes acute lymphoblastic leukemia with Ewing sarcoma. Hepatosplenomegaly was found in 35.90% of patients while cardiotoxicity was observed in 34 (11.41%) patients (Table 2). The consort diagram (Fig. 1) displays that 180 (60.40%) patients meet the ACMG criteria and were referred to genetic counselor. Among them, 145 (48.65%) patients have consanguineous parents, 29 (9.73%) patients have a family history of LS-associated cancer in 1st and 2nd-degree relatives and 6 (2.01%) patients have sibling with a childhood cancer.

**Table 2:** Anthropometric characteristics and history of patients with ALL

Characteristics	Number	Percentage (%)
Mean age at diagnosis ±SD	6.62 ± 3.5	
Gender		
Male	215	72.14%
Female	83	27.85
Parental consanguinity		
Yes	151	50.67
No	147	49.32
Family History of Cancer		
Yes	66	22.14
1st degree	6	9.09
2nd degree	47	71.21
3rd degree	20	30.30
No	232	77.85
No of primary cancer		
One	299	99.66
Two	1	0.34
Type of ALL		
Pre B-ALL	257	86.24
Pre T-ALL	41	13.75
WBC count		
< 50,000	285	95.63
≥ 50,000	13	4.36
Hepatospleenomegaly		
Yes	107	35.90
No	191	64.09
Cardiotoxicity		
Yes	34	11.41
No	264	88.59

### Impact of parental-consanguinity and history on the characteristics of ALL

The parental consanguinity was observed in 151 indexed patients included in the study. Based on the age of patients, WBC count, hepatosplenomegaly and cardiotoxicity and sub-types of ALL the data were divided into two groups: consanguineous and non-consanguineous. The statistical analysis showed that the parental consanguinity had a protective impact when compared with the age of the patient. No significant association was observed with other variables. The patient with the two primary cancers has a history of parental consanguinity (Table 3). In our cohort, 66 (22.14%) patients have a family history of cancer in first, second and third-degree relatives. The impact of familial history of cancer was also analysed with the age of the patient, WBC count, hepatosplenomegaly and cardiotoxicity and types of ALL the data. Except for cardiotoxicity, no significant association was observed with other variables. Familial history of cancer increases 2.46 times the risk of the outcome of cardiotoxicity (Table 4).

**Table 3:** Impact of parental consanguinity on the characteristics and type of ALL patients with no associated disorder

Characteristics	Parental consanguinity (***n*** = 51)	No parental consanguinity (***n*** = 147)	Odds ratio (95% CI)	***P***-value
Age of the patient				
< 10	105 (69.53%)	123 (83.67%)	0.44 (0.25–0.77)	0.00*
≥ 10	46 (30.46)	24 (16.32%)		
WBC count				
< 50,000	144 (95.36%)	141 (95.91%)	0.87 (0.28–2.66)	0.82
≥ 50,000	7 (4.63%)	6 (4.08%)		
Hepatospleenomegaly				
Yes	56 (37.08%)	51 (38.77%)	1.11 (0.69–1.78)	0.66
No	95 (62.91%)	96 (65.30%)		
Cardiotoxicity				
Yes	15 (9.93%)	19 (12.92%)	0.74 (0.36–1.52)	0.41
No	136 (90.06%)	128 (87.07%)		
Sub-type of ALL				
Pre-BALL	132 (87.42%)	125 (85.03%)	1.22 (0.63–1.36)	0.55
Pre-TALL	19 (12.58%)	22 (14.96%)		
BCR-ABL translocation	1 (0.66%)	0	–	–
Two primary cancers in the affected child	1 (0.66%)	0	–	–

**Table 4:** Impact of familial history on the characteristics and type of ALL patients with no associated disorder

Characteristics	Familial history of disease (***n*** = 66)	No familial history of disease (***n*** = 232)	Odds ratio (95% CI)	***P***-value
Age of the patient				
< 10	49 (74.24%)	179 (77.15%)	0.85 (0.45–1.60)	0.62
≥ 10	17 (25.75%)	53 (22.84%)		
WBC count				
< 50,000	65 (98.48%)	220 (94.82)	3.54 (0.45–27.78)	–
≥ 50,000	1(1.51%)	12 (5.17)		
Hepatospleenomegaly				
Yes	21 (31.81%)	86 (37.06%)	0.79 (0.44–1.41)	0.43
No	45 (68.18%)	146 (62.93)		
Cardiotoxicity				
Yes	13 (19.69%)	21 (21.98%)	2.46 (1.15–5.24)	0.02*
No	53 (80.30%)	211 (90.94)		
Sub-type of ALL				
Pre-BALL	57 (86.36%)	200 (86.20%)	1.01 (0.45–1.24)	0.86
Pre-TALL	9 (13.63%)	32 (13.79%)		
BCR-ABL translocation	0 (0.00)	1 (0.43%)	–	–
Two primary cancers in the affected child	0 (0.00)	1 (0.43%)	–	–

### Family history association with parental consanguinity

In our data, the ALL patients with family history of cancer with parental consanguinity (24.50%) were more than non-parental consanguinity (19.73%) however, no significant impact of parental consanguinity was observed on a family history of cancer. We also assessed the impact of parental consanguinity on the degree of family history of cancer. The statistical analysis showed no significant impact of parental consanguinity on the first, second, third and first and second degree of family history however, the odds ratio of first degree relative [4.14 (CI = 0.45–37.60)] was higher as compared to the second 1.18 [(0.62–1.20)], third [1.72 (0.60–4.90)], first and second-degree relatives [1.31 (0.71–1.43)] with cancer. The association of the number of relatives of cancer with the parental consanguinity did not show any significant association as well (*p*-value < 0.05) (Table 5).

**Table 5:** Impact of parental consanguinity on family history of cancer and number of relatives with cancer

	Parental consanguinity		OR (95% CI)	***P***-value
	YES (***N*** = 151)	NO (***N*** = 147)		
**Family history of cancer**				
Yes	37 (24.50%)	29 (19.73%)	1.32 (0.76–1.29)	0.32
No	114 (75.49%)	118 (80.27%)		
First degree relatives	4 (10.81%)	1 (3.45%)	4.14 (0.45–37.60)	–
Second degree relatives	25 (67.57%)	22 (68.96%)	1.18 (0.62–2.20)	0.61
Third degree relatives	10 (27.03%)	6 (20.69%)	1.72 (0.60–4.90)	0.30
First and second degree relatives	29 (78.37%)	22 (75.86%)	1.31 (0.71–2.43)	0.38
**Number of relatives with cancer**				
1	141(93.37%)	142 (96.59%)	0.49 (0.16–1.48)	0.20
≥ 1	10 (6.62%)	5 (3.40)		

## Discussion

This study assesses the high risk individual for hereditary cancer susceptibility syndrome (HCSS) in childhood acute lymphoblastic leukemia and to our knowledge, this is the first study conducted in the Pakistani population using the ACMG practice guidelines. We demonstrated that 60.40% of the paediatric ALL patients fulfils the criteria for the assessment of HCSS (Fig. 1). A previous study conducted in the highly consanguineous population of Saudi Arabia using the ACMG practice guideline has identified 40% of cancer patients with HCSS that is lower than the Pakistani population [12]. This may be due to the fact that our study population focus only the childhood ALL while, the study patients in Jastaniah and co-workers, comprise all the cancers diagnosed in multi-institution in Saudi Arabia. Knapke and co-workers also reported 29% of childhood cancer survivors meet the criteria for the HCSS however different criteria were established for the assessment of HCSS that comprise of medical and familial history of cancer [11].

ACMG guidelines limit the parental consanguinity for the leukemia and brain tumours as these cancers have been reported to be associated with constitutional mismatch repair deficiency (CMMRD) syndrome [25]. In our cohort, 48.65% of patients were eligible for genetic counselling solely due to the presence of parental consanguinity (Fig. 1). We also studied the association between parental consanguinity and family history with age, WBC count chemotherapy related toxicity and subtype of ALL. The results showed that age at the diagnosis of disease, hepatosplenomegaly and subtype of ALL was comparable among consanguineous and non-consanguineous patients, however; parental consanguinity showed significantly protective impact age at diagnosis of disease (Table 3). This is in concordance with the previous studies performed in highly consanguineous populations of the Middle East that suggests that consanguinity confers a protective effect on the development of adult cancers at an early age [26–28]. In the previous studies, different germline genetic variations have been associated with the chemotherapy-related cardiotoxicity [29, 30]. We also found that cardiotoxicity to be comparable in patients having a family history of cancer; compared with patients without a family history of cancer, our study showed that a familial history of cancer asserts an increased risk of toxicity in ALL patients (Table 4). At present, the outcome of chemotherapy-related toxicities has not been utilized by the ACMG guidelines as a criterion for the assessment of HCSS, and our study suggests to be factored into the assessment guidelines.

It is interesting that despite high consanguinity (50.67%) in the population only 29 patients shown to have of a family history of cancer in 1st and 2nd-degree relatives moreover, 2 (0.66%) patients have associated syndrome that is Down syndrome and only 1 (0.34) patient has second primary cancer (Table 2). This reflects that HCSS or CMMRD is not much prevalent in our population; otherwise these numbers would be higher. It may also be attributed due to short the follow-up duration of the patient. It can also be concluded that the high frequency of consanguinity alone does not increase the risk of family history and the results were consistent when compared with the 1st, 2nd and 3rd degree of relatedness and number of relatives with cancer (Table 5). Despite a high parental consanguinity in the Pakistani population, the 5 year prevalence of leukemia was reported (8.56 per million from 2015 to 2020) less than in some non-Asian regions; Australia and New Zealand (11.3 per million between 2003 and 2007) where parental consanguinity exists < 1% [31]. Thus, it affirms that consanguinity alone does not increase the risk of leukemia. Moreover, the previous studies indicated the autosomal recessive pattern for inheritance of cancer predisposition syndromes was associated with consanguinity, and the inheritance of disease cannot be found in the subsequent generation unless consanguinity is more complex or extended [32], in this situation, ACMG guidelines might not be ideal to show such a correlation.

The results of our study are in concordance with the recommendations made by the Jastaniah et al. suggesting the score based criteria based on multifactorial genetic risks factors, the toxicology profile and physical features along with the personal and family history of cancer for the identification of high risk individuals for HCSS especially, for the highly consanguineous populations [12]. Genetic evaluation of large number of patients may not be feasible especially for developing country; therefore, to manage the resources, “Genetic testing for all children with cancer” approach is needed to be re-evaluated. To assess the prevalence of the germline mutations or variation related to hereditary cancer susceptibility, the population-based screening using high throughput techniques is needed to be performed. The possible limitations of the study include self-reporting bias related to the family history of disease and lack of availability of pathology reports of the relatives having a history of cancer. The self-reporting bias was reduced by using a structured standardized self-reporting questionnaire conducted by the trained individual. Various studies have been performed to analyse the validity of self-reporting of family history of cancer and found that the sensitivity ranges from 0.78 to 0.90 [33, 34], moreover the recall bias is expected to be lowered due to the extended family support system of Pakistani culture.

## Conclusion

Results of our cross-sectional study in acute lymphoblastic leukeima have identified 60.40% of high risk patients with hereditary cancer susceptibility syndrome (HCSS) by using ACMG guidelines. Parental consanguinity was the leading criteria for the identification, however; the findings of our study underscore revisiting this criterion especially in highly consanguineous populations. Population-based screening of mutations and variation related to hereditary susceptibility is needed to be performed in the Pakistani population that aims to contribute to the development of a genetic-screening tool for the early assessment of cancer.

## Data Availability

The datasets used and/or analysed during the current study are available from the corresponding author on reasonable request.
